# Roles of Specialized Pro-Resolving Lipid Mediators in Cerebral Ischemia Reperfusion Injury

**DOI:** 10.3389/fneur.2018.00617

**Published:** 2018-07-31

**Authors:** Ping Yin, Yafen Wei, Xu Wang, Mingqin Zhu, Jiachun Feng

**Affiliations:** ^1^Department of Neurology and Neuroscience Center, First Hospital of Jilin University, Changchun, China; ^2^First Department of Neurology and Neuroscience Center, Heilongjiang Provincial Hospital, Harbin, China

**Keywords:** cerebral ischemia reperfusion, inflammation, resolution, specialized pro-resolving lipid mediators, stroke

## Abstract

Ischemic stroke contributes to ~80% of all stroke cases. Recanalization with thrombolysis or endovascular thrombectomy are currently critical therapeutic strategies for rebuilding the blood supply following ischemic stroke. However, recanalization is often accompanied by cerebral ischemia reperfusion injury that is mediated by oxidative stress and inflammation. Resolution of inflammation belongs to the end stage of inflammation where inflammation is terminated and the repair of damaged tissue is started. Resolution of inflammation is mediated by a group of newly discovered lipid mediators called specialized pro-resolving lipid mediators (SPMs). Accumulating evidence suggests that SPMs decrease leukocyte infiltration, enhance efferocytosis, reduce local neuronal injury, and decrease both oxidative stress and the production of inflammatory cytokines in various *in vitro* and *in vivo* models of ischemic stroke. In this review, we summarize the mechanisms of reperfusion injury and the various roles of SPMs in stroke therapy.

## Overview of cerebral ischemic reperfusion injury

Stroke is one of the most common causes that leads to morbidity and mortality all over the world. In the acute phase of ischemic stroke, treatments should focus on saving penumbral tissue as much as possible. There are two main recanalization methods: (1) intravascular thrombolysis with recombination tissue-type plasminogen activator (rt-PA) (1, 2), and (2) endovascular thrombectomy (3). It has been demonstrated that recanalization improves the clinical prognosis in acute stroke patients. However, in some cases, severe side effects, such as fatal edema or intracranial hemorrhage, are reported after ischemia reperfusion (4). Moreover, animal studies showed that reperfusion after a long ischemic period could result in a larger damage size than the volume that corresponds to the permanently occluded vessel (5, 6). Therefore, even though reperfusion may reduce infarct size and improve clinical outcomes, it may also exacerbate cerebral injury via a process named “cerebral ischemia reperfusion injury (CIRI)” (4). Many studies support the idea that oxidative stress and inflammation are involved in CIRI, and ultimately lead to apoptosis and cell death (7, 8).

### Cellular events involved in CIRI

#### Endothelial cells (ECs)

CIRI is characterized by increased blood-brain barrier (BBB) permeability. ECs are critical structures that make up the BBB, a strong mechanical barrier in the brain that prohibits the transcellular movement of large molecules. ECs are tightly interconnected through specific proteins called tight junctions, which function to seal the inter-endothelial spaces, thereby inhibiting the delivery of immune cells and hydrophilic molecules. Under ischemic conditions, excessive accumulation of reactive oxygen metabolites may cause ECs to swell and detach, leading to impaired BBB function, protein extravasation, and interstitial edema (9). Furthermore, BBB breakdown induces ECs to produce selectins, integrins, and pro-inflammatory cytokines and chemokines, such as interleukin (IL)-1β, tumor necrosis factor (TNF)-α, IL-6, and prostaglandins. The released pro-inflammatory mediators affects the microvasculature in the injury site, where leukocytes attach to the ECs and cause reduced blood supply and hypoxia (9). Leukocytes activation including the production of cytokines, which then further upregulating the expression of adhesion molecules on the surface of both ECs and leukocytes. The increased expression of leukocyte adhesion molecules and the increased production of reactive oxygen species, leading to the peroxidation of cellular membrane components, further lead to the element of vascular permeability and vasogenic edema (10). Therefore, a vicious cycle of inflammation finally occurs.

#### Microglial cells

Microglial cells are resident macrophages in the brain. Traditionally, activated microglia are classified into two subtypes: M1 and M2 phenotypes, with M1 phenotypes having pro-inflammatory and M2 phenotypes playing anti-inflammatory roles (11, 12). Evidence shows that microglial cells are activated quickly after ischemia onset and produce a series of pro-inflammatory mediators, such as reactive oxygen species (ROS) (13), IL-1, IL-6, TNF-α, and matrix metalloproteinase (MMP)-9, which can be toxic to neurons. It has been found that these events precede leukocyte infiltration into the brain. Thus, early microglial activation plays a detrimental role by increasing BBB permeability, therefore, contributing to the increased number of leukocytes infiltration to the brain (14, 15). In fact, inhibiting microglial activation in the early phase of CIRI protects the brain against injury by maintaining BBB integrity (14). Furthermore, inhibiting M1 microglial migration in the hyper-acute phase of transient middle cerebral artery occlusion (MCAO) significantly attenuates infarct volumes and improves neurological outcomes (16). In later phases of CIRI, blood monocytes/macrophages infiltrate the brain. Microglia and macrophages share some common features, but many investigations have shown that there are markers that distinguish microglia from hematogenous infiltrating macrophages (17). Activated microglia/macrophages could play a protective role in later phases of CIRI by triggering neutrophil apoptosis and phagocytosis of apoptotic neutrophils, thereby preventing the release of toxic molecules into the nearby tissue. This is a crucial step in resolution of inflammation and preventing further tissue damage of CIRI (18). The protective role of microglia/macrophages may also be mediated by their ability to produce various neurotrophic factors and anti-inflammatory cytokines, such as fibroblast growth factor, transforming growth factor (TGF)-β1, IL-4, IL-10, and IL-13, which are involved in ending inflammation and initiating tissue repair (12, 19, 20). A recent study found that microglia depletion exacerbated post-ischemia inflammation and brain injury via augmented inflammatory mediators produced by astrocytes. Microglia can restrict the ischemia-induced astrocytic response (21).

#### Astrocytes

After cerebral ischemia, astrocytes undergo reactive astrogliosis, characterized by swelling in morphology, enhanced expression of glial fibrillary acidic protein, and upregulated Calcium signaling (22). Astrocytes upregulate major histocompatibility complexes and inflammatory mediators, such as pro-inflammatory cytokines (IL-6 and IL-1β), chemokines [CXC-chemokine ligand (CXCL) 1, CXCL10, CXCL12, and monocyte chemoattractant protein 1 (MCP-1)], and inducible nitric oxide synthase (iNOS). Although astrocytes may be harmful due to their hyper reactivity to toxic injuries and glial scar, astrocytes also perform multiple homeostatic functions for neurovascular unit survival and maintenance (23). For instance, astrocytic glycogen stores maintain neuronal metabolism under hypoglycemia (24, 25). Furthermore, astrocytes modulate the ion buffering (26), uptake toxic neurotransmitters (27, 28), synthesize neuroprotective mediators (29), control cerebral blood flow (CBF) via the release of vasoactive substances, such as prostaglandin E2 and epoxyeicosatrienoic acids (30), transport water, release antioxidant molecules (31), and likely participate in adult neurogenesis (32).

#### Neutrophils

Neutrophils infiltrate the injury site early following CIRI (33). They are recruited from the blood to injury site through specific bindings with chemokines, for instance CXCL1, CXCL2, and CXCL3. A recent study has shown that blocking neutrophil-specific chemokine receptors, such as CXC-chemokine receptor (CXCR), reduces ischemic brain injury (34). Scavenger receptors, such as cluster of differentiation 36 (CD36), may also play important roles in neutrophil accumulation after ischemic CNS injury (35). Neutrophils respond to damage-associated molecular patterns (DAMPs) via binding to toll-like receptors (TLRs). Once activated by inflammatory mediators, neutrophils upregulate their surface adhesion receptors, such as CD15 and CD11b, to promote their adherence to ECs and their migration into inflamed tissue (36). The mechanisms underlying neutrophil-mediated damage in CIRI include neutrophil sludging, causing microvascular hypoperfusion, excessive production of ROS, release of pro-inflammatory cytokines and chemokines, elastase, and MMPs, and increased adhesion molecule expression. In one study, there were three-fold more neutrophils in permanent MCAO compared to transient MCAO, suggesting that ongoing hypoxia is a major stimulus for immune cell infiltration (37). Although evidences have suggested that preventing neutrophil infiltration into the ischemic tissue inside the brain can minimize infarct size (38, 39), some studies also suggest that the number of neutrophils in an ischemic brain does not predict stroke severity (40). Hence, the role of neutrophils in stroke is more complicated than what we currently know.

#### Platelets

The inflammatory roles of platelets have recently been highlighted (41, 42). During inflammation, ECs express intercellular adhesion molecule (ICAM)-1, platelet selectin (P-selectin), endothelial selectin (E-selectin) as well as vascular cell adhesion molecule (VCAM) (43), causing platelets to adhere to and activate ECs. Platelets express CD154 (also known as CD40L) on their surface and secrete IL-1β. In turn, platelet CD154 can cause ECs to secrete adhesion molecules including E-selectin, ICAM-1, and VCAM-1, as well as chemokines such as MCP-1 and anti-inflammatory cytokines IL-8 (44). IL-1β release further increases EC permeability and recruits leukocytes to attach to ECs (45). Together, this augments inflammation and activates inflammatory cells, such as monocytes and neutrophils. Platelet P-selectin and CD154 are respectively recognized by P-selectin glycoprotein ligand 1 of monocytes and CD40 of neutrophils, leading to the formation of platelet-leukocyte aggregates and thereby contributing to the innate immune response (46). In addition, platelet activation can release serotonin and chemokine ligand (CCL) 5, which are known to mediate T cell activation and differentiation. Platelets also produce many pro-inflammatory chemokines and cytokines, and may also promote inflammation by releasing microparticles (47–49). Platelets are major sources of circulating microparticles (50). Microparticles contain proteins including P-selectin, chemokines, such as CCL5, and cytokines, such as IL-1β (51). Microparticles also contain different forms of RNAs, such as microRNA (miRNA), small non-coding RNAs that play important roles in post-transcriptional regulation of gene expression. Platelets may affect the nearby cells by transferring miRNA (52, 53). For example, miR-320b transferred into ECs can decrease ICAM-1 expression (52). In the same way, miR-126-3p, can be taken up by macrophages, resulting in an increased phagocytic phenotype (53).

#### T cells and B cells

As BBB permeability increases, DAMPs may invade the brain and function as antigenic molecules for the immune system. After stroke onset, an infiltration of lymphocytes may also directly contribute to brain injury (54). Studies utilizing ischemic stroke mice models have demostrated that the numbers of both T cells and antigen-presenting cells increase in delayed phase (3–7 days) of post-reperfusion, suggesting that antigen presentation and T cell responses may play a role in the pathophysiology of ischemic stroke (55, 56). CD4^+^ helper, CD8^+^ cytotoxic, and γδT cells play detrimental roles in various stroke models (57, 58). CD4^+^ T cells produce pro-inflammatory cytokines, such as interferon (IFN)-γ, and CD8^+^ T cells secrete perforin/granzymes (59, 60), all of which could cause neuronal death and worsen stroke outcomes. It is thus unsurprising that, in mice, CD4^+^ helper/CD8^+^ cytotoxic T cell deficiency results in smaller infarct size, less infiltrating leukocytes, and better prognosis after transient MCAO (57). γδT lymphocytes are involved in ischemic brain injury by producing the pro-inflammatory molecule IL-17 (58, 61). In stroke, IL-17 and TNF-α synergistically stimulate astrocytes to secrete chemokines that are attractive for neutrophils, such as CXCL-1, leading to the infiltration of neutrophil, and thus increased damage of brain tissue (62). Furthermore, T regulatory cells (Tregs) might play a protective role in ischemic brain injury by producing the anti-inflammatory cytokines IL-10 as well as TGF-β (63) and down-regulating the neutrophil production of MMP-9, protecting the BBB (64, 65). In addition, the anti-CD28 antibody CD28SA was proved to improve neurological function after experimental ischemic stroke by increasing the number of Treg cells (66). On the contrary, there have been suggestions that Treg cells could be harmful in the early phase of ischemic brain injury for the reason that they may cause dysregulation of immune system and vascular malfunction (67, 68).

In the weeks to months after stroke, immunoglobulin synthesis was found in the cerebrospinal fluid (CSF) of ischemic stroke patients (69), suggesting B cell were activated as a result of brain damage. Consistent with this finding, it has also been found that activated B cells could have an impact on the cognitive function and recovery (70).

#### Dendritic cells (DCs)

DCs serve as antigen presenting cells in the brain that process antigenic materials and present them on their surface to immune cells. Under normal conditions, DCs cannot be found inside the brian (71). It has been demonstrated that DCs are present in the parenchyma of inflamed brains as early as 1 h after ischemia in an MCAO rat model (72). In line with this findings, Gelderblom et al. demonstrated that DCs were observed inside the brain together with other type of immune cells after CIRI (55). Importantly, results from experimental studies prove that DC infiltration after ischemia stroke worsens the clinical outcomes (73). Granulocyte-colony stimulating factor (G-CSF) mediates the migration of DCs after transient MCAO and, thus, inhibiting G-CSF could attenuate cerebral infarct volume and inflammation (73). Furthermore, DCs that present in the ischemic brain injury site can activate T cells, initiating a long-lasting immune response and worsening the clinical outcomes.

#### Mast cells (MCs)

MCs locate in various parts of in the brain, including the cerebral cortex, thalamus, and diencephalic parenchyma. Importantly, MCs that locate in the perivascular positions have the capability to quickly respond to stimuli, and produce of a series mediator such as vasodilatory, pro-inflammatory mediators and proteolytic molecules. Therefore, MCs can play an important role in the defense of various stimuli. Several mediators synthesized by MCs can affect stroke outcomes. TNF-α which comprises 25% of the MC granule, enhances BBB permeability and increases T cell infiltration, proliferation, function, and cytokine production (74). MC-derived endothelin, endoglin, and MMP-9 increase neutrophil infiltration, BBB leakage, and edema after reperfusion in a transient MCAO mouse model (75). MC-derived gelatinase contributes to early ischemic BBB disruption and edema formation (76). MC-derived IL-6 contributes to increased brain granulocytes, macrophage activation, infarct size exacerbation, and brain swelling (77).

### Molecular events involved in CIRI

Free radicals and pro-inflammatory mediators are quickly produced from the damaged tissue after ischemic stroke. These mediators can cause cerebral ECs to synthesize adhesion molecules, increase adhesion and trans-endothelial migration of leukocytes from the periphery. Infiltrating leukocytes can then release more cytokines and chemokines, which produce free radicals that complement and activate MMPs. This further amplifies inflammation by promoting local immune cell activation and leukocyte infiltration, eventually leading to BBB disruption, brain edema, and neuronal death.

#### The role of free radicals in CIRI

There are two major classes of free radicals: ROS and reactive nitrogen species (RNS). During thrombolysis, reperfusion may cause increased ROS and RNS production, both of which are mediators of neurotoxicity and BBB breakdown.

##### ROS

ROS consists of active species, including hydroxyl radicals, superoxide anion radicals, peroxide (H_2_O_2_), hydrogen, and singlet oxygen, among others. Under normal conditions, ROS have critical biological functions and function as redox signaling molecules. However, under pathological conditions, such as CIRI, excessive ROS are produced. In various ischemia/reperfusion (I/R) organs, xanthine oxidase, ROS are synthesized by the enzymatic activity of NOS and nicotinamide adenine dinucleotide phosphate (NADPH). In CIRI, the mitochondria are the main source of ROS (78). I/R cause electron leakage and excessive production of free radical in the mitochondrion. Over abundant free radicals can cause oxidative injury to the mitochondrial respiratory chain, metabolizing enzymes further increase the electron leakage and free radical release (79, 80). Moreover, free radicals damage mitochondrial membrane structures (81), and increase mitochondrial permeability transition pore opening (82), leading to impairment of membrane potential and more oxidative stress (80). Increased mitochondrial permeability also increases the release of pro-apoptotic mediators into the cytoplasm (83). Moreover, I/R-induced damage also impairs mitochondrial dynamics and mitophagy, thereby affecting the quality control of the mitochondrial network (84, 85). Eventually, mitochondrial dysfunction leads to increased apoptosis. ROS also directly contribute to neuronal death by oxidizing proteins, damaging DNA, and inducing lipid peroxidation.

##### RNS

Two common RNS that are well documented in CIRI are nitric oxide (NO) and peroxynitrite (ONOO^−^). A low-concentration of NO produced by endothelial NOS (eNOS) acts as an indispensable messenger to regulate pre- and/or post-synaptic activities (86), as well as maintaining vascular physiological functions (87), CBF (88), and inflammatory responses (89), whereas high-concentrations of NO produced from iNOS and neuronal NOS can trigger cell death and BBB leakage during I/R injury (90). iNOS activation upregulates autophagy of vascular ECs and increases apoptosis during CIRI (91). During CIRI, NO is synthesized together with superoxide (O2^−^·) and quickly interacts with O2^−^· at a diffusion-limited rate to produce ONOO^−^. In physiological conditions, ONOO^−^ can directly react with thiols, or the radical products of ONOO^−^ decomposition may indirectly oxidize other cellular components, such as lipids, proteins, and DNA. However, excessive ONOO^−^ triggers inflammation, lipid membrane peroxidation, and mitochondrial dysfunction (92), subsequently exacerbating BBB disruption and brain dysfunction (93). Plasma 3-nitrotyrosine, which is often used as an ONOO^−^ footprint, has a positive correlation with brain ischemic severity in stroke patients. RNS-mediated autophagy/mitophagy may play an indispensable role in CIRI. ONOO^−^-mediated autophagy could also induce tight junction protein degradation (94).

#### The role of complement proteins in CIRI

Complement proteins play a key role in the inflammatory reaction following CIRI. In ischemic stroke, BBB disruption allows circulating complement proteins to enter the CNS; further, complement proteins can also be locally produced by CNS resident cells. Due to excessive glutamate and ionic imbalance, CIRI results in a depletion of cellular energy resources, as well as an increase in ROS release, apoptotic and necrotic cell death, and excitotoxic insults. These pathological processes result in the activation of complement proteins via different pathways including the classical, alternative and extrinsic protease pathways. All of these pathways are involved in the formation of membrane attack complexes (MACs). The other two products that emerge following complement protein activation are opsonins (C1q, mannose-binding lectin (MBL) and C3b/i C3b/C3d) and anaphylatoxins (C3a and C5a). The alternative pathway is consistently active, and hydrolyzed C3 can be placed on the surface of immune cells that can lead to subsequently C3 activation. The classical pathway is activated by apoptotic cells or through recognition by Clq. The extrinsic protease pathway is activated through the specific binding of carbohydrate molecules by ficolins or MBL. Activation of these pathways leads to activation of C3. C3 cleavage generates C3a and C3b, the latter is cleaved further to generate membrane-bound iC3b, C3d, C3dg and opsonins, which are recognized by their specific receptors on the immune cells. C3 cleavage also result in C5 cleavage, generating soluble C5a and membrane-bound C5b, the later starts the terminal pathway and leads to the formation of MAC that directly lyse the cells and stimulates cells to produce pro-inflammatory mediators. The anaphylatoxins C3a and C5a recruit and activate leukocytes. The complement opsonins promote microglial phagocytosis and release IFN-γand other cytokines (95). Signaling of C3d through complement receptor 2 inhibits neuronal progenitor cell proliferation (96).

Infarct development was strongly correlated with robust complement protein activation (97). The presence complement molecules including Clq, MBL, C3, C4d, C5a, and C9 has been observed in ischemic brain sites (98, 99) and increased levels of C3, C4d, C5a, and C5b-9 have also been found in the plasma of stroke patients (100). CD59, expressed by astrocytes and microglia, is a MAC inhibitory protein. Exacerbated pathology and behavior deficits have been found in CD59 knockout ischemic stroke mice (101). In line with these findings, experiments using other complement related genetic models proved the pathological roles of Clq (102), C3 (103, 104), and C5 (105) in stroke. Genetic MBL deficiency is protective in clinical and experimental severe cerebral I/R injuries (106), but not in mild cerebral ischemia (107). It has been demonstrated that intravenous rt-PA dramatically upregulates complement cascade activation in ischemic brains, and pharmacologic inhibition of complement proteins protects brains against the adverse effects of rt-PA thrombolysis in stroke (108). These studies have led many researchers to focus on inhibiting complement proteins to alleviate stroke injuries (109–112), including depletion of complement proteins, suppressing complement-driven cellular recruitment, neutralizing MBL, and inhibiting complement protein activation, among others. Other investigations have targeted down-stream mediators of the activated complement system. Anaphylatoxin C3a was thought to have neuroprotective and neurotrophic properties (113). C3a regulated astrocytes response to ischemia and increased their capacity to react to ischemic stress (114). Studies have also shown that applying specific C3a receptor agonists protects against intestinal I/R injury by inhibiting neutrophil mitigation (115). In subacute to chronic phases of ischemic stroke, C3a–C3a receptor signaling stimulated post-stroke synaptogenesis and axonal plasticity, and intranasal treatment with C3a receptor agonists improved functional recovery (116). C5a interacts with its canonical receptor C5aR1 and initiate the complement-mediated inflammatory processes. Its inhibition is generally thought to mitigate acute cerebral I/R injury (117). Since complement proteins are also important for immune surveillance and homeostatic activities, many therapies that systemically inhibit complement systems have potential risks. Nonetheless, complement inhibitors that target specific sites are under investigation (118).

#### The role of MMPs in CIRI

MMPs are responsibe for protein digestion and extracellular matrix (ECM) turnover and belong to a group of zinc-binding endopeptidases. There are 23 different types of MMPs in humans, which are classified according to their structural similarity (119). Microglia are major sources of MMPs (39). Activated MMPs can be found immediately after ischemia onset and last for 5 days (39, 119). MMPs mediate basal lamina protein disruption by causing BBB hyperpermeability, leukocyte extravasation, cerebral edema, and hemorrhagic transformation (HT). Although different types of MMPs act differently, there is accumulating evidence that MMP-2, MMP-9, and MMP-12 are involved in the pathogenesis of BBB disruption and cerebral edema formation during ischemic stroke.

MMP-9-deficient mice had smaller ischemic volumes compared with controls in a permanent focal cerebral ischemia mouse model (120). Some clinical studies have suggested that the levels of circulating MMP-9 are significantly correlated to disease severity and infarct volume in the hyperacute phase (121, 122), as well as late hemorrhagic infarction incidence between 5 and 7 days after stroke onset (123). A recent study showed that higher MMP-9 levels in the serum of patients with acute ischemic stroke is correlated with the severity of clinical symptoms; Therefore, MMP-9 may serve as an important biomarker for ischemic stroke patent (124). Furthermore, rt-PA was shown to upregulate MMP-9 levels *in vivo* in a rat model of stroke (125), emphasizing the impact of MMP-9 activation in rt-PA-associated side effects, such as HT.

The role of MMP-2 in ischemic stroke is more complex. It has been shown previously that the levels of MMP-2 are lower in the acute phase of stroke mice model. However, the levels of MMP-2 in the serum of stroke patients are not correlated to infarct size or stroke disability (126). Actually, MMP-2 activity has been shown increased in the latter phase of in ischemic mice model (119) and the MMP-2 levels are higher in ischemic stroke patients in the recovery phase (126). A recent study using a non-human primate MCAO model sampled CSF and found that MMP-2 and MMP-13, but not MMP-9, correlate to the CSF/serum albumin ratio, a sign of BBB permeability (122).

MMP-12 expression increases as early as 1 h after permanent focal cerebral ischemia (127) and can persist for 14 days after I/R (128). Elevated MMP-12 after ischemic stroke disrupts the BBB by degrading tight junction proteins (127). MMP-12 also contributes to ischemic brain cell apoptosis, infarct volume enlargement, myelin basic protein degradation, and demyelination (128). Additionally, MMP-12 can activate other MMPs, such as pro-MMP-1 and pro-MMP-9 (129). Thus, MMP-12 enhances the proteolysis cascade. Studies show that plasma MMP-12 levels are associated with stroke severity and elevated MMP-12 might predict poor outcomes (130).

**Figure 1:**
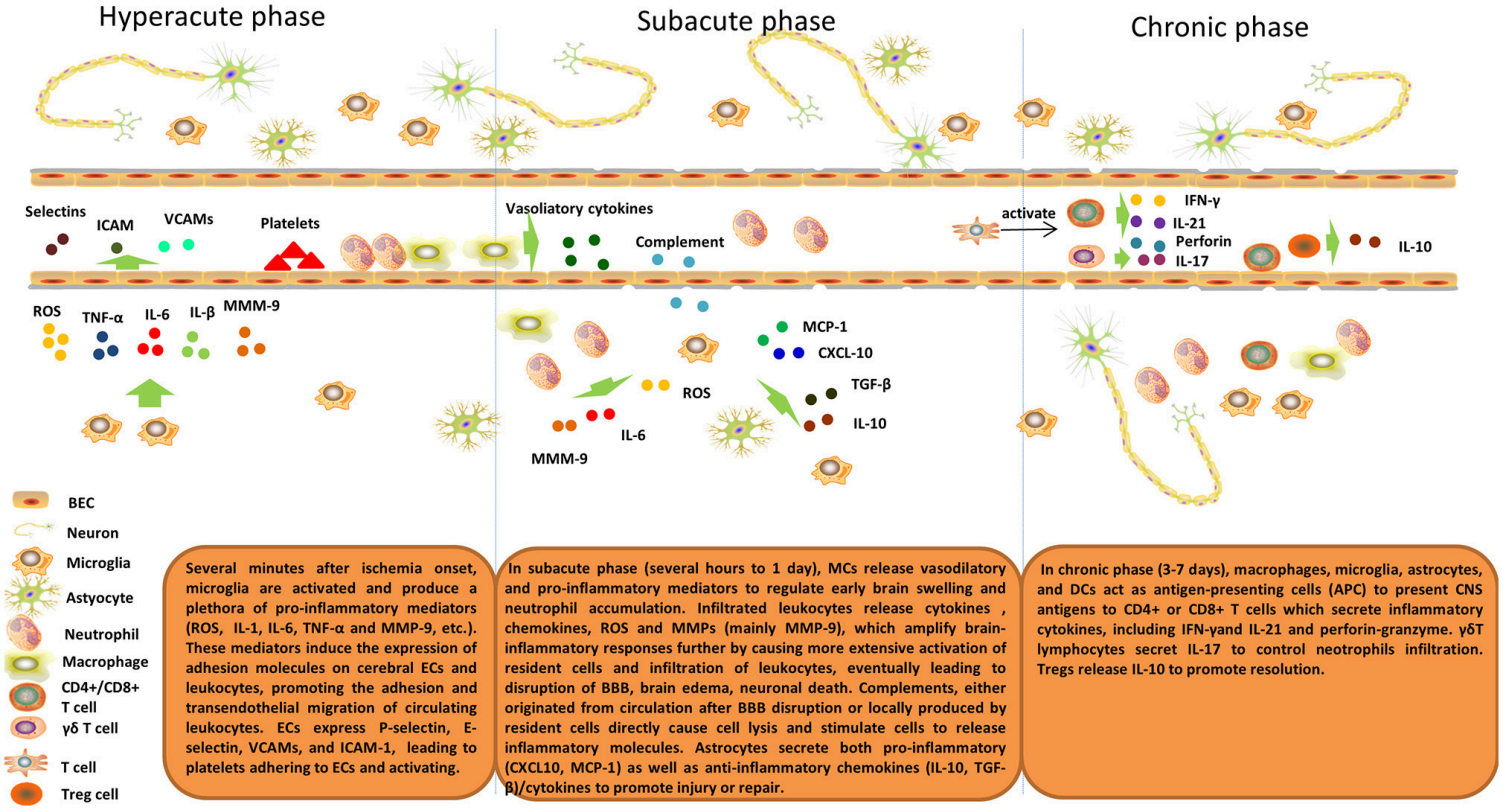
Inflammatory response after cerebral ischemia. Brain post-ischemic inflammatory responses are characterized by innate immune activation followed by adaptive immune activation. Microglial cells are activated within minutes of ischemia onset and produce a plethora of pro-inflammatory mediators (ROS, IL-1, IL-6, TNF-α, and MMP-9, etc.). These mediators induce expression of adhesion molecules on cerebral ECs and leukocytes and, thus, promote adhesion and transendothelial migration of circulating leukocytes. ECs express P-selectin, E-selectin, VCAMs, and ICAM-1, which lead to platelets adhering to and activating ECs. In the subacute phase (hours to 1 day), MCs release vasodilatory and pro-inflammatory mediators to regulate early brain swelling and neutrophil accumulation. Infiltrating leukocytes release cytokines, chemokines, ROS and MMPs (mainly MMP-9), which amplify brain-inflammatory responses further by causing more extensive activation of resident cells and infiltration of leukocytes, eventually leading to disruption of the BBB, brain edema, and neuronal death. Complements, either originating from the circulation after BBB disruption or locally produced by resident cells directly cause cell lysis and stimulate cells to release inflammatory molecules. Astrocytes secrete both pro-inflammatory (CXCL10, MCP-1) and anti-inflammatory chemokines (IL-10, TGF-β)/cytokines to promote injury or repair. In the delayed phase (3–7 days), macrophages, microglia, astrocytes, and DCs act as antigen-presenting cells (APCs) to present CNS antigens to CD4^+^ or CD8^+^ T cells that secrete inflammatory cytokines, including IFN-γ and IL-21 and perforin-granzyme. γδT lymphocytes secrete IL-17 to control neutrophil infiltration. Tregs release IL-10 to promote resolution.

The inflammatory response after cerebral ischemia is described in Figure 1.

## Resolution of inflammation in CIRI

A transient inflammatory reaction exists within the first week after ischemic stroke (131). Ultimately, the inflammation subsides, and inflamed brain tissue returns to homeostasis. Resolution of inflammation is an active and highly regulated biochemical process mediated by a group of lipid mediators, termed specialized pro-resolving mediators (SPMs). Four classes of SPMs have been discovered, including resolvins, lipoxins, protectins, and maresins. The complete stereochemistry of each SPM is known (132). The biological functions of SPMs include cessation of neutrophil infiltration, decreased production of pro-inflammatory mediators, increased uptake of apoptotic neutrophils and cellular debris, and promotion of macrophage transformation from the M1 to M2 phenotype, among others (49, 133).

### Synthesis of SPMs

In the acute phase of the inflammatory response, free polyunsaturated fatty acids (PUFAs) were released from the membrane by the activity of phospholipase enzymes [e.g., cytosolic phospholipase A2 (cPLA2)]. SPM are derived from PUFAs, which including arachidonic acid (AA) that belongs to omega-6 fatty acid and eicosapentaenoic acid (EPA), and docosahexaenoic acid (DHA) that belong to omega-3 fatty acids. The crucial enzymes involved in this synthesis are lipoxygenases (LOX), cyclooxygenases (COX), and cytochrome P450 monooxygenases (CYP450). SPMs are generated by leukocytes, structural cells, some organs, and tissue during the resolution of inflammation (134) and their production is ultimately controlled by the vagus nerve (135, 136).

Lipoxins are biosynthesized from AA. Three main lipoxin synthesis pathways have been reported. In the first pathway, Lipoxin A4 (LXA4) and lipoxin B4 (LXB4) are produced by the oxygenation of AA by 15-LOX and 5-LOX, and then by enzymatic hydrolysis in mucosal tissues (137). In the second pathway, LXA4 is produced by 5-LOX in blood vessels and LXB4 is synthesized by 12-LOX in platelets. The third pathway is aspirin-triggered pathway, aspirin changes COX-2 activity by increasing the acetylation of COX-2, leading to the production of a new lipoxin analog termed aspirin-triggered lipoxins (138). Inactivation of lipoxin catalysis by the enzyme 15-prostaglandin dehydrogenase can result in the synthesis of a series of stable lipoxin analogs (139) that retain all of the biological functions of lipoxins.

Two groups of resolvins exist: the the D-series, derived from DHA and E-series, derived from EPA (140). Two resolvin E1 (RvE1) formation pathways have been reported. The first is the acetylation of COX-2 at Ser^516^ by aspirin and 5-LOX (141). The second is oxidation by CYP450 followed by 5-LOX oxidation (142). RvE2 is derived from EPA via the 18R-hydroxyeicosapentaenoic acid and 5S-hydroperoxide, which is 5-LOX dependent (143). Afterwards, the 5S-hydroperoxide is directly converted to RvE2 without epoxidation process. Unlike RvE1 and RvE2, the formation of a third member, RvE3, does not require 5-LOX (144). DHA can be converted to 17-hydroperoxide intermediate (17-H(p)DHA) in the presence of 15-LOX, which can further give rise to D-series resolvins (RvDs). The hydroperoxide 5-LOX can be converted to 7S, 8S-epoxide by epoxidation. 7S, 8S-epoxide can then further converted to RvD1 or RvD2 enzymatic hydrolyzation (145). RvD3–D4 as well as aspirin-triggered 17R D-series resolvins (AT-RvDs) have also been discovered and their stereochemical structures have been reported recently (146, 147).

Protectins are also biosynthesized from DHA. DHA is firstly to a 17S-hydroxyperoxide-containing intermediate converted by lipoxygenase, and then the intermediate is engulfed by leukocytes to give rise to protectin D1 (PD1 or NPD1) (148). Besides, aspirin-triggered protectin D1 (AT-NPD1), has been uncovered as well (149).

The maresins (MaRs) synthesis pathway is started by the 14-lipoxygenation of DHA to generate a 14S-hydro(peroxy)-4Z,7Z,10Z,12E,14S,16Z,19Z-docosahexaenoic acid intermidate and then 13S,14S-e MaR by the enzymatic activity of 12-LOX. 13S,14S-e MaR converted to MaR1 by enzymatic hydrolyzation or epoxy hydration, to MaR2.

### The roles of SPMs in CIRI

As discussed above, inflammation plays an important role in CIRI, so it is plausible that resolving inflammation would be a promising therapeutic target for CIRI. In animal models of ischemic stroke, SPMs and their aspirin-triggered forms, are endogenously produced (150–152) and have protective roles.

#### The effects of SPMs on ECs

Vascular ECs play an important role in modulating the passage of molecules and cells from the circulation cross the vessel wall during the inflammation. Therefore, vascular permeability and EC activation is critical to resolve inflammation in CIRI. Moreover, ECs are involved in SPM production and can express receptors that SPMs can directly interact with during acute phase of inflammatory response (153). For example, ECs express ALX/FPR2 (formyl-peptide receptor type 2 or LXA4 receptor; termed Fpr2/3 in mice) and G-protein coupled receptor (GPR) 32, which are receptors both for LXA4 and RvD1. Upon binding to these receptors, SPMs can regulate leukocyte recruitment and adhesion. It has been demonstrated that LXA4 downregulates VCAM-1, E-selectin (154), and ICAM-1 (155), and increases prostacyclin production, which then counter-regulates leukocyte–EC interactions and platelet activation (156). Moreover, LXA4 has further EC-specific effects, such as downregulating NADPH oxidase, which decreases ROS generation (157). An LXA4 analog reduces brain injury by improving BBB function, inhibiting MMP-9 expression, and upregulating tissue inhibitor of metalloproteinase-1 (TIMP-1) protein (158). Resolvins also have the ability to regulate microvasculature permeability. RvD1 protects the human EC barrier against endotoxin-induced impairment (159) and aspirin-triggered-RvD1, reducing the endothelial barrier permeability in an acute lung injury mice model (160).

#### The effects of SPMs on polymorphonuclear leukocytes (PMNs)

After ischemia, leukocyte infiltration, as well as ROS and pro-inflammatory mediator production, results in tissue damage. Therefore, inhibiting leukocyte accumulation or removing leukocytes altogether, is crucial for brain protection during ischemia. Various ischemia models in different organs have supported the idea that SPMs inhibit leukocyte accumulation and promote phagocytosis of apoptotic leukocytes. In a mesenteric artery I/R mouse model, an LXA4 analog detached leukocytes from endothelium cells by binding to FPR (161) and also diminished PMN infiltration into lungs after hind limb I/R (162–164). RvD1 has been reported to reduce fibrosis and the accumulation of leukocytes, as well as enhancing cardiac function after in experimental mice model of myocardial infarction (MI) (152). Biosynthesis of RvDs and protectins has been found after I/R in the kidneys. Furthermore, treatment with RvDs prior to ischemia decreases leukocyte infiltration and tissue fibrosis (151). In the context of CIRI, RvD1 and PD1 are also protective, as they reduce the number of infiltrating leukocytes and prevent NF-κB and COX-2 activation in neurons (150). In a mouse hind limb I/R model, resolvins protect against leukocyte recruitment into the lungs (165, 166). RvEl has potent effects on leukocytes by decreasing migration, inhibiting rolling, enhancing CCR5 expression, and downregulating NF-κB pathway signaling (167). In a mouse renal pedicle I/R model, PD1 reduces kidney PMN influx and amplifies renoprotective heme-oxygenase(HO)-1 protein and mRNA expression (168).

#### The effects of SPMs on macrophages

Efferocytosis, or the clearing of dead cells, is essential for tissue to successfully resolve the inflammatory response. Cardiac function impairment has been found in acute MI mice deficient in Mertk and/or Mfge8, which are the receptors of efferocytosis. This is caused by an abrogation of efferocytosis rather than a change in circulating or tissue-resident macrophage/monocyte infiltration (169, 170). A study using a bilateral common carotid artery occlusion (BCCAO)/reperfusion mouse model has demonstrated that LXA4 analogs can enhance efferocytosis, thereby promoting a resolution of inflammation (171). Macrophage/microglia survival and phagocytosis in a highly oxidative environment after CIRI is of critical importance. It has been demonstrated that RvD1, upon binding to ALX/FPR2, has protective effects on macrophages during efferocytosis from apoptosis induced by oxidative stress, by down-regulating NADPH oxidase and the reducing the levels of apoptotic proteins such as Bcl-x and Bcl-2 (172). During efferocytosis, SPM production increases, further enhancing debris and apoptotic cell clearance (173).

SPMs are also thought to modify macrophage phenotype from pro-inflammatory M1 to anti-inflammatory M2 by binding to GPR32 (174). The shift in phenotype toward M2 is associated with reparative and anti-inflammatory functions. RvD1 has been proved protective I/R injury model of liver by promoting M2 polarization of macrophage and promoting efferocytosis (175). It has also been shown to induce M2 macrophage phenotypes to resolve adipose tissue inflammation (176). It has been demonstrated that RvD2 administration increases macrophage polarization to the M2 phenotype in the arterial walls of a murine model of arterial neointima formation (177). MaR1 has also been shown to stimulate the M1 to M2 phenotype transformation besides its pro-resolving bio functions and tissue restorative abilities (178).

As we know, ECs, PMNs, and microglia/macrophages are primary sources of inflammatory molecules during CIRI. The pro-inflammatory mediators such as cytokines, chemokines as well as adhesion molecules, which contribute to leukocyte infiltration, oxidative stress, BBB disruption, and neurotoxic substance release, among others. Anti-inflammatory cytokines inhibit pro-inflammatory cytokines and stimulate tissue repair during inflammation resolution. SPMs can increase anti-inflammatory mediators and decrease pro-inflammatory mediators. In a superior mesenteric artery I/R mouse model, an LXA4 analog decreases vascular permeability, leukocyte influx, and hemorrhage in the intestines, as well as inhibiting reperfusion-induced remote injury to the lungs by activating ALX/FPR2 for IL-10 production (179). In a bilateral kidney I/R mouse model, an LXA4 analog alleviates I/R injury by reducing PMN infiltration and pro-inflammatory cytokine (*IL-1, IL-6*) and chemokine (*growth regulated oncogene-1 [GRO1]*) mRNA levels, and increasing renal mRNA levels of *(SOCS)-1* and *SOCS-2 that are suppressors of cytokine signaling* (180). Further, by using oligonucleotide microarray and bioinformatics, Kieran et al. found that the LXA4 analog is renoprotective since it modifies changes in the mRNA levels of many families of harmful molecules, including pro-inflammatory cytokines, cellular adhesion molecules, and proteases (181).

#### The effects of SPMs on oxidative stress and metabolism

As discussed above, oxidative stress plays an important role in the pathogenesis of CIRI. SPMs can attenuate CIRI by acting as potent antioxidants and mediators. In a spinal cord I/R rabbit model, LXA4 improves neurological function and reduces cell apoptosis, partly by decreasing MDA levels and increasing superoxide dismutase (SOD) activity (182). In different I/R experimental models, the mechanisms by which LXA4 attenuates oxidative stress includes downregulating GRP-78 (78 kDa glucose-regulated protein) and caspase-12 (183), activating the Keap1/Nrf2 (Ketch-like ECH-associated protein 1/nuclear respiratory factor 2) (184) and Nrf2 (185) pathways, and acting as an agonist of peroxisome proliferator-activated receptor (PPAR)γ (186). MaR1 suppresses free radicals by activating HO-1 pathway mediated by Nrf-2 in a left pulmonary hilum I/R mouse model (187).

**Figure 2:**
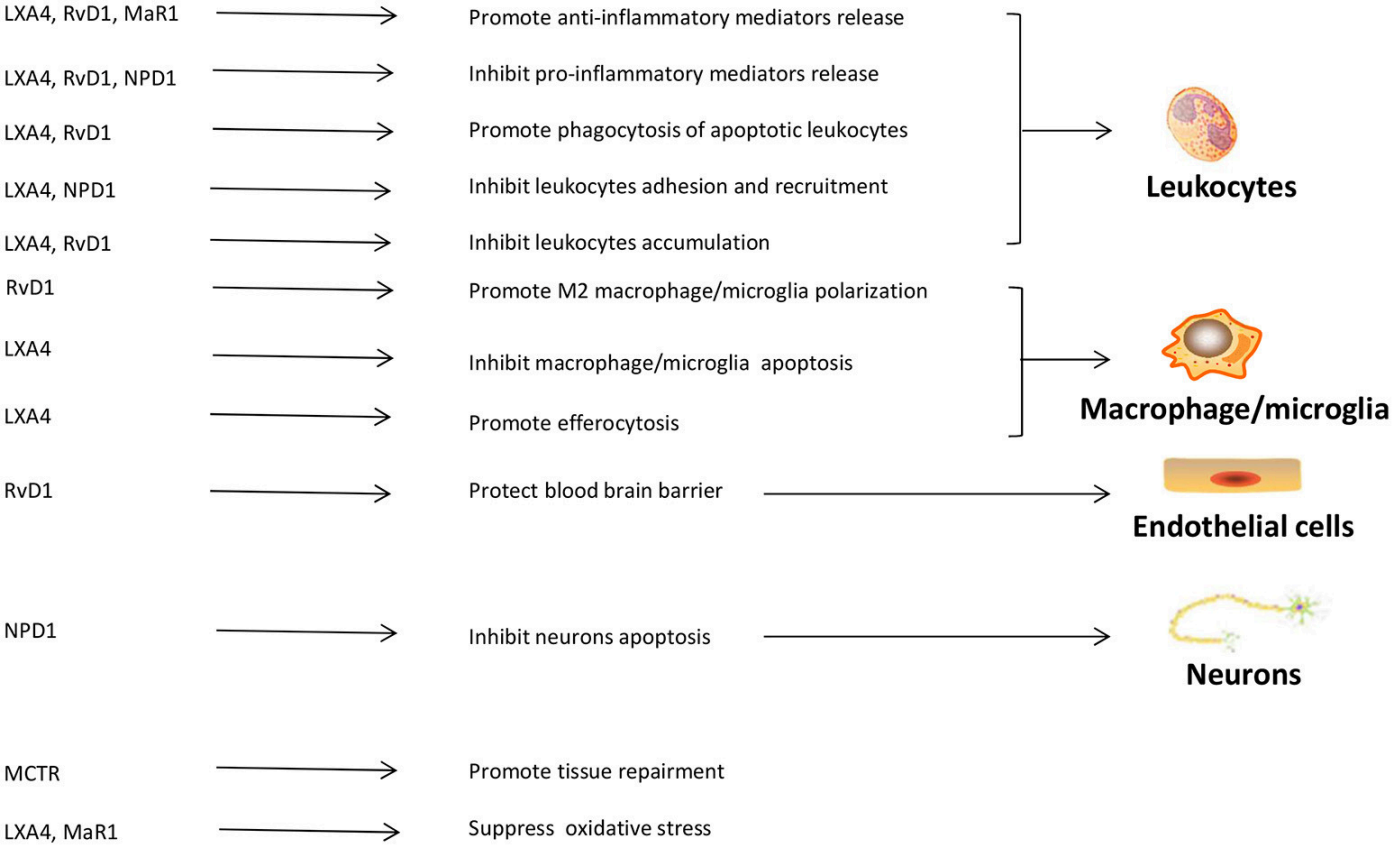
The roles of SPMs in cerebral ischemia reperfusion injury. SPMs can display their protective roles through interacting with leukocytes, macrophages/microglia, vascular endothelial cells, and neurons in cerebral ischemic reperfusion injury. The main functions of SPMs in cerebra ischemic reperfusion injury include regulating leukocytes adhesion, infiltration and apoptosis, mediating inflammatory mediators release, adjusting macrophages/microglia polarization, protecting blood brain barrier, inhibiting neuronal apoptosis, promoting tissue restitution, suppressing oxidative stress. LXA4, lipoxin A4; RvD1, resolving D1; MaR1, maresin1; NPD1, protectin D1; MCTR, maresin conjugates in tissue regeneration.

In CIRI, SPMs may improve energy metabolism disorders to protect tissue from injury. RvD1 has been shown protective for mitochondrial in I/R lung model, by increasing Na^+^-K^+^-ATPase activity, retune the balance of ATP/ADP ratio, and decreasing apoptosis (188). LXA4 attenuates metabolic disturbance in MI by upregulating Na^+^-K^+^-ATPase expression (189). LXA4 also upregulates Cx43 expression to prevent arrhythmogenesis (189). The roles of each SPM on cells and moleculars in CIRI are summarized in Figure 2.

## Promoting inflammation resolution to treat I/R injury

The discovery that the resolution of inflammation is a highly regulated process is crucial, because this knowledge has led to investigations of new therapeutics with selective targets that will safely resolve inflammation without compromising host defense (190). Each SPM has potent pro-resolving functions that are fundamental to inflammation resolution. Current experiments are investigating if targeting these pro-resolution functions can be useful in the treatment of CIRI (Table 1).

**Table 1:** Studies of SPMs in I/R injury experiment models.

**SPMs**	**Disease model**	**Action**	**Mechanisms**	**References**
Lipoxin and its analogs	MCAO/reperfusion model	Ameliorating BBB dysfunction.	Inhibiting MMP-9 and increasing TIMP-1 protein expression.	(158)
		Regulating neutrophil-platelet aggregate (NPA) formation, inhibiting cerebral microvasculature reactivity.	Through binding with ALX/FPR2	(191)
		Inhibiting 5-lipoxygenase translocation and leukotrienes biosynthesis	Through ERK signal transduction pathway.	(192)
		Suppressing PMNs infiltration and lipid peroxidation levels, inhibiting microglia and astrocytes activation, reducing pro-inflammatory cytokines and up-regulating anti-inflammatory cytokines.	Inhibiting NF-κB activation.	(193)
		Reducing oxidative stress.	Activating Nrf2 pathway and its nuclear translocation, as well as HO-1 expression and GSH synthesis.	(194)
	Mesenteric artery I/R model	Provoking adherent leukocytes detachment from endothelium.	Through binding with ALX/FPR2.	(161, 195)
		Decreasing vascular permeability, leukocyte influx, and hemorrhage in intestine, suppressing TNF-α production.	Associated with enhanced IL-10 production.	(179)
		Reducing oxidative stress	Through activating Keap1/Nrf2 pathway.	(184)
	Hindlimb I/R model	Inhibiting PMNs infiltration in remote organs.		(162–164)
	Bilateral common carotid artery occlusion (BCCAO)/reperfusion model	Reducing the number of rolling cells, adherent leukocytes and activated microglial cells, increasing plasma MCP-1 and IL-6 levels.	Through binding with FPR2/3.	(171)
	Bilateral kidney I/R model.	Inhibiting PMNs infiltration, reducing IL-1β, IL-6, and GRO-1 expression.	Modulation of renal mRNA levels for the suppressors of cytokine signaling SOCS-1 and SOCS-2.	(180)
		Modifing many pathogenic mediators expression, including cytokines, growth factors, adhesion molecules, and proteases.		(181)
	Spinal cord I/R model.	reducing cell apoptosis and MDA levels, increasing SOD activity.		(182)
	Left anterior descending coronary artery I/R model.	Inhibiting neutrophil activation, attenuating myocardial oxidative stress and inhibition of apoptosis, attenuating metabolic disturbance.	downregulation of GRP-78 and caspase-12, upregulating Na^+^-K^+^-ATPase expression.	(183, 189)
	Permanent MCAO.	Decreasing infarct volume and neurological deficit.	Through agonist of PPARγ.	(186)
	Primary cultured astrocytes exposed to OGD/recovery	Reducing oxidative stress.	Through activating Nrf2 pathway.	(185)
	Celiac artery I/R model.	Preventing mucosal injury induced by either cyclooxygenase or lipoxygenase inhibitors.		(196)
Resolvins	Left anterior descending coronary artery occlusion model	1) Discontinuing neutrophil priming in spleen and LV post-MI. 2) Stimulating macrophages clearance from infarcted area. 3) Reducing ECM gene expression and collagen deposition.	Reducing pro-fibrotic genes and decreasing collagen deposition.	(152)
		Decreasing infarct size and attenuating Depression-like symptoms.		(197)
	Left coronary artery I/R model. H9c2 cells exposed to hypoxia /reoxygenation	Increasing cell viability and decreased apoptosis.	Activation of pro-survival pathways (Akt and ERK1/2).	(198)
	Bilateral kidney I/R model. Swine kidney epithelial cells treated with H_2_O_2._	RvDs reduced kidney interstitial fibrosis. RvDs and PD1 reduced infiltrating leukocytes numbers and activation of macrophages.	Blocking TLR.	(151)
	hind LIMB I/R model	Inhibiting PMNs infiltration in remote organs.		(165, 166)
	Hepatic portal triad I/R model	Inhibiting PMNs infiltration, enhancing M2 macrophage polarization and efferocytosis.		(175)
		Attenuating IL-6, TNF-α, and myeloperoxidase levels, reducing apoptosis.	Increasing phosphorylation of Akt.	(199)
	Lung hilum I/R model	Improving energy metabolism disturbance, protecting mitochondrial structure and function and decreasing apoptosis.	Increasing ATP, glycogen content and Na^+^-K^+^-ATPase activity, balancing the ratio of ATP/ADP.	(188)
		Inhibiting complement, immunoglobulin, and PMNs activation and inflammatory factors expression.	Down-regulating TLR4/NF-κB.	(200)
Protectins	MCAO/reperfusion model	Improving neurological scores, reducing infarction volumes and edema.	Through activation of Akt and p70S6K pathways.	(201, 202)
	MCAO/reperfusion model, human neural progenitor cells exposed to IL-1β	Reducing leukocytes infiltration, preventing pro-inflammatory gene expression.	Inhibiting NF-κB activation and cyclooxygenase-2 expression.	(150)
	MCAO/reperfusion model, retinal pigment epithelial (RPE) cells exposed to UOS	Protecting cells against death induced by cerebral ischemia and UOS.	Upregulating ring finger protein 146 which facilitated DNA repair.	(203)
	Renal pedicles I/R model, glomerular mesangial cells exposed to serum starvation.	Reducing leukocytes infiltration.	Amplifing Reno protective HO-1 protein and mRNA expression.	(168)
Maresins and MCTR	Lung hilum I/R model.	Suppressing oxidative stress.	Through activation of the Nrf-2-mediated HO-1 signaling pathway.	(187)
	MCAO/reperfusion model.	Mitigating inflammation.	Inhibiting NF-κB activation.	(204)
	Hindlimb I/R model.	Inhibiting PMNs infiltration, regulating cell proliferation, and tissue repayment.	Up-regulating Ki67 and Roof plate-specific spondin3 expression.	(205)

*ADP, adenosine diphosphate; ATP, adenosine triphosphate; ALX/FPR2, synonym formyl peptide receptor; BBB, blood brain barrier; ECM, extracellular matrix; ERK, extracellular signal-regulated kinase; GRO-1, growth regulated oncogene-1; GRP-78, glucose-regulated protein; GSH, glutathione; HO-1, haeme oxygenase-1; IL, interleukin; I/R, ischemia/reperfusion; LV, left ventricle; MCAO, middle cerebral artery occlusion; MCP-1, monocyte chemoattractant protein; MCTR, maresin conjugates in tissue regeneration; MDA, malondialdehyde; MI, myocardial infarction; MMP-9, metalloproteinase-9; NF-κB, nuclear factor-κB; Nrf2, nuclear factor erythroid 2-related factor 2; OGD, oxygen-glucose deprivation; PD1, protectin D1; PMN, polymorphonuclear leukocyte; PPARγ, mediated by transcription factor peroxisome proliferator-activated receptors gamma; Rv, resolvin; SOCS, suppressors of cytokine signaling; SOD, superoxide dismutase; TIMP-1, tissue inhibitors of metalloproteinase-1;TLR, toll-like receptor; TNF-α, tumor necrosis factor-α; UOS, uncompensated oxidative stress*.

### LXA4

LXA4 binds with high affinity to the GPR termed ALX/FPR2, which is expressed on various cellular types, such as leukocytes, ECs, fibroblasts, microlgia, macrophage, and neuronal cells (206). Activation of this receptor could promote a resolution of inflammation and tissue repair. Exogenous introduction of LXA4 in Fpr2/3^+/+^mice attenuates inflammation in I/R injury, while this result cannot be observed in Fpr2/3^−/−^ mice, demonstrating the protective role of Fpr2/3 in I/R injury modles (191, 195). Consistent with these findings, studies in CIRI rats have found that LXA4 is also neuroprotective, reducing leukocyte infiltration and astrocyte activation, decreasing TNF-α and IL-1β production, and reducing leukotrienes, as well as extracellular signal-regulated kinase (ERK) phosphorylation (192, 193). It has been demonstrated that LXA4 reduces infarct size and improves neurological scores, partially by inducing Nrf2 synthesis and its translocation into the nucleus, as well as increasing HO-1 and glutathione (GSH) synthesis; thus, combating increased oxidative stress (194). In addition, the PPAR agonist rosiglitazone has been shown to be neuroprotective by increasing LXA4 and reducing leukotriene B4 (LTB4) in experimental stroke (186). Besides its neuroprotective effects, LXA4 may also attenuate the side effects of drugs that treat cerebrovascular diseases. In a celiac artery I/R rat model, LXA4 prevents against cyclooxygenase or lipoxygenase inhibitors induced injury of celiac mucosal (196).

### Resolvins

RvD1 binds to and activates human GPR32 and ALX/FPR2, while recombinant GPR32 can be active by RvD3 and RvD5 (207). RvD2 activates a novel GPR termed DRV2/GPR18, and RvD2-DRV2 interactions could stimulate macrophage phagocytosis (166, 208). RvDs may inhibit PMN activation and decrease their infiltration, as well as enhancing macrophage phagocytosis of apoptotic cells/bacteria (190). RvD1 is endogenously produced in response to I/R injury in kidneys, inhibition of leukocyte accumulation, and TLR-mediated macrophage activation (151). Moreover, RvD1 resolves inflammation and enhances ventricular function after MI (152). In rats, RvD1 inhibits inflammatory responses following liver I/R injury (199) and this protective effect is related to a shift to M2 phenotype of macrophage and enhanced efferocytosis induced by ALX/FPR2 activation. The underlying mechanisms mediating the protection of I/R injury might involve the inhibition of complements, activation of immunoglobulin and neutrophils, downregulation of TLR4/NF-κB, and decreased expression of pro-inflammatory factors (200). Inflammation resolution involves a specific miRNA, regulated by SPM receptors (209, 210). RvD1-GPR32 increases miR-208 and the anti-inflammatory cytokine IL-10, and downregulates miR-219, decreasing LTB4 via the regulation of 5-LOX (209). In a left anterior descending coronary artery I/R rat model, RvD1 not only decreases infarct size, but also attenuates depression-like symptoms, suggesting that RvD1 might be involved in neurotransmitter secretion (197).

RvE1 binds to GPR ChemR23 and LTB4 receptor 1 (BLT1). ChemR23 is highly expressed in dendrites and macrophages (141). RvE1 has been shown to decrease infarct size in a rat model of MI/reperfusion injury by decreasing apoptosis in cells induced by hypoxia (198). RvE1 stimulates macrophage phagocytosis of apoptotic leukocytes, decreases the pro-inflammatory cytokines IFN-γ as well as IL-6 production (211), inhibits DC migration, and reduces pro-inflammatory cytokine release (141) via phosphoprotein-mediated signaling (212). The pro-inflammatory lipid mediator LTB4 binds to BLT1 and promotes leukocyte survival. RvE1 competes with LTB4 and blocks the binding of LTB4 to BLT1 to increase the apoptosis of leukocytes and efferocytosis (213). It has also been reported that ChemR23 expression is restricted to naive M1, but not M2, macrophages (214). The authors also found that treating M1 macrophages that express ChemR23 with RvE1 enhanced the phagocytosis of microbial particles and increased IL-10 transcription, resulting in a resolution-type macrophage that is different from the anti-inflamatory M2 phenotype.

### NPD1

NPD1 is generated in rodents after ischemia and plays an important role in PMN decrement (150). Bazan and colleagues have demonstrated the beneficial roles of NPD1 in the CNS. NPD1 reduces oxidative stress, thus improving cell survival. NPD1 also has effect on microglia cells in ocular disease models (215). AT-NPD1 is endogenously generated by aspirin and DHA treatment in the cerebral stroke models. Synthetic 17R-NPD1 has been shown reduced the brain edema and the infarct size and reduces the neurological deficiency (201). 17R-NPD1 derived from aspirin-triggered DHA reduces human PMN infiltration, enhances microphage efferocytosis (216), improves neurological scores, and reduces total lesion volume and brain edema, as computed by T2-wighed image (T2WI) (201). In an MCAO/reperfusion rat model, NPD1 significantly reduces the infarct sizes in aged rats via activation of the Akt and p70S6K pathways (202). Furthermore, NPD1 up-regulates ring finger protein 146, which facilitates DNA repair and protects cells against death induced by cerebral ischemia (203).

### Maresin

Intracerebroventricular injection of MaR1 has been shown to significantly reduce infarct volumes and neurological defects, protecting the neurons from injury in a CIRI mouse model, by reducing the pro-inflammatory responses and inhibiting the activation of NF-κB p65 and its translocation to the nucleus (204). In a hindlimb I/R mouse model, maresin conjugates in tissue regeneration (MCTR) protects the lungs and spleen from tissue damage mediated by leukocytes and increases cell growth and tissue restoration in the lungs (205). A recent study has found that platelets express the SPM receptors GPR32 and ALX. MaR1 could enhance platelet aggregation, spreading and suppressing the release of pro-inflammatory and pro-thrombotic mediators; therefore, MaR1 may be used as a new class of antiplatelet agents (217).

## Conclusions and future perspectives

CIRI can worsen patient outcomes through excessive inflammation. However, neuronal damage following CIRI is often irreversible; therefore, there is an unmet clinical need to explore novel therapeutic options to resolve the aggressive inflammatory state after cerebral ischemic insults to limit the irreversible neural damage. Dietary n-3 supplements are widely used, but clinical trials with n-3 fatty acids have yielded mixed results (218). The lack of success may be because aging patients and those with obesity-associated diseases may have difficulty converting PUFAs into SPMs (219). The biosynthesis pathways for both omega-3 and omega-6 fatty acids are complex and involve competition for enzymes to produce various bioactive mediators without pro-resolving actions. Thus, SPMs may be more potent and biologically relevant than fatty acids, their nutritional precursors (220, 221). The new challenge ahead is if the novel SPMs that stimulate inflammation resolution can be harnessed to treat I/R injury. Stable synthetic mimetics to endogenous SPMs and synthetic SPM receptor agonists are under development for various chronic inflammatory disease models (133); however, the side effects accompanying these need to be investigated further (222). It is our hope that the results from these studies will provide new stroke treatments by controlling resolution and downstream mechanisms of inflammatory responses.

## Author contributions

PY, XW, and YW wrote the manuscript. MZ and JF reviewed the topic and established a basic framework for the manuscript.

### Conflict of interest statement

The authors declare that the research was conducted in the absence of any commercial or financial relationships that could be construed as a potential conflict of interest. The reviewer DP-G and handling Editor declared their shared affiliation.
